# Supplementary material for: Multi‐dataset identification of innovative feature genes and molecular mechanisms in keratoconus
Source: J Cell Mol Med. 2024 Sep 19;28(18):e70079. doi: 10.1111/jcmm.70079 (PMC11412914; doi:10.1111/jcmm.70079)
Supplement: Supplementary file 2 — Material S2. [file JCMM-28-e70079-s002.zip › 7.GSEA analysis/4.MYRF/KEGG_PRIMARY_IMMUNODEFICIENCY.html]

Details for gene set KEGG\_PRIMARY\_IMMUNODEFICIENCY[GSEA]

|  || Dataset | MYRF.group.cls#h\_versus\_l |
| Phenotype | group.cls#h\_versus\_l |
| Upregulated in class | h |
| GeneSet | KEGG\_PRIMARY\_IMMUNODEFICIENCY |
| Enrichment Score (ES) | 0.23900093 |
| Normalized Enrichment Score (NES) | 0.6554389 |
| Nominal p-value | 0.936345 |
| FDR q-value | 1.0 |
| FWER p-Value | 1.0 |
Table: GSEA Results Summary

  

Fig 1: Enrichment plot: KEGG\_PRIMARY\_IMMUNODEFICIENCY      
 Profile of the Running ES Score & Positions of GeneSet Members on the Rank Ordered List

  

| SYMBOL | TITLE | RANK IN GENE LIST | RANK METRIC SCORE | RUNNING ES | CORE ENRICHMENT || 1 | CD19 | na | 21 | 1.070 | 0.2263 | Yes |
| 2 | JAK3 | na | 4412 | 0.241 | 0.1508 | Yes |
| 3 | TNFRSF13C | na | 5257 | 0.215 | 0.1720 | Yes |
| 4 | ZAP70 | na | 5428 | 0.210 | 0.2115 | Yes |
| 5 | IL7R | na | 5912 | 0.195 | 0.2390 | Yes |
| 6 | CD4 | na | 10052 | 0.095 | 0.1399 | No |
| 7 | BLNK | na | 10233 | 0.092 | 0.1541 | No |
| 8 | TNFRSF13B | na | 10434 | 0.088 | 0.1670 | No |
| 9 | CD3D | na | 11300 | 0.073 | 0.1576 | No |
| 10 | CD79A | na | 11413 | 0.072 | 0.1696 | No |
| 11 | ICOS | na | 11751 | 0.067 | 0.1741 | No |
| 12 | UNG | na | 12152 | 0.061 | 0.1756 | No |
| 13 | DCLRE1C | na | 12246 | 0.060 | 0.1856 | No |
| 14 | CD3E | na | 15808 | 0.020 | 0.0871 | No |
| 15 | AICDA | na | 16059 | 0.017 | 0.0835 | No |
| 16 | BTK | na | 17142 | 0.007 | 0.0539 | No |
| 17 | RAG1 | na | 17262 | 0.006 | 0.0518 | No |
| 18 | RFXANK | na | 18895 | -0.009 | 0.0067 | No |
| 19 | AIRE | na | 19057 | -0.011 | 0.0044 | No |
| 20 | RFXAP | na | 20879 | -0.032 | -0.0413 | No |
| 21 | IKBKG | na | 21687 | -0.042 | -0.0556 | No |
| 22 | TAP1 | na | 22885 | -0.058 | -0.0779 | No |
| 23 | CIITA | na | 22934 | -0.058 | -0.0669 | No |
| 24 | CD8B | na | 23062 | -0.061 | -0.0577 | No |
| 25 | IGLL1 | na | 23279 | -0.064 | -0.0504 | No |
| 26 | ADA | na | 23859 | -0.073 | -0.0515 | No |
| 27 | PTPRC | na | 24862 | -0.091 | -0.0612 | No |
| 28 | RFX5 | na | 25004 | -0.093 | -0.0455 | No |
| 29 | CD8A | na | 25192 | -0.097 | -0.0304 | No |
| 30 | TAP2 | na | 28437 | -0.173 | -0.0871 | No |
| 31 | IL2RG | na | 29736 | -0.215 | -0.0790 | No |
| 32 | LCK | na | 29935 | -0.221 | -0.0379 | No |
| 33 | RAG2 | na | 30001 | -0.223 | 0.0076 | No |
| 34 | CD40LG | na | 30708 | -0.251 | 0.0404 | No |
| 35 | CD40 | na | 32572 | -0.356 | 0.0621 | No |
Table: GSEA details [plain text format]

  

Fig 2: KEGG\_PRIMARY\_IMMUNODEFICIENCY      
 Blue-Pink O' Gram in the Space of the Analyzed GeneSet

  

Fig 3: KEGG\_PRIMARY\_IMMUNODEFICIENCY: Random ES distribution      
 Gene set null distribution of ES for **KEGG\_PRIMARY\_IMMUNODEFICIENCY**
